# Gender differences in occupational exposure to carcinogens among Italian workers

**DOI:** 10.1186/s12889-018-5332-x

**Published:** 2018-03-27

**Authors:** Alberto Scarselli, Marisa Corfiati, Davide Di Marzio, Alessandro Marinaccio, Sergio Iavicoli

**Affiliations:** Department of Occupational and Environmental Medicine, Epidemiology and Hygiene, Italian Workers’ Compensation Authority (INAIL), Rome, Italy

**Keywords:** Gender disparities, Exposure assessment, Occupational health, Surveillance system, Prevention database, Carcinogenic agents

## Abstract

**Background:**

Many carcinogenic chemicals are still used or produced in several economic sectors. The aim of this study is to investigate differences in occupational exposure patterns to carcinogens by gender in Italy.

**Methods:**

Information about the most common carcinogens recorded in the Italian occupational exposures database (SIREP) for the period 1996–2015 was retrieved. Descriptive statistics were calculated for exposure-related variables (carcinogenic agent, occupational group, economic activity sector, and workforce size). The chi-square(χ^2^) test was used to verify differences between genders, and logistic regression analysis was performed to evaluate the association between gender and risk of having higher exposure levels, after adjusting for age. Concurrent exposures to multiple carcinogens were investigated using the two-step cluster analysis.

**Results:**

A total of 166,617 exposure measurements were selected for 40 different carcinogens. Exposed workers were only in a small proportion women (9%), and mostly aged 20–44 years (70%) in both genders. Women were more likely to be exposed than men to higher levels for several carcinogens even after correction for age at exposure, and the exposure level was significantly (*p* < 0.01) associated with occupation, economic sector and workforce size. The five main clusters of co-exposures identified in the entire dataset showed a differential distribution across economic sectors between genders.

**Conclusions:**

The exposures to occupational carcinogens have distinguishing characteristics in women, that are explained in part by work and job segregation. Because of the presence of high-exposed groups of female workers in many industrial sectors, further research and prevention efforts are recommended.

## Background

Most of epidemiological studies investigating occupational exposure to carcinogens are conducted in the male working population, representing the majority of exposed workers [1]. Women’s employment in Italy has increased by almost 50% over the last 35 years, although the female participation rate in the labor market (ratio between workforce and working age population), despite growing, is still below the European Union (EU) average [2]. National estimates commonly report smaller percentages and lower levels of exposure to carcinogens among female workers, but it has been argued that exposures considered are usually biased towards industrial occupations and exposures where measurements are available, underrepresenting, for example, the exposure in the service sector [3]. On the other hand, the relevant gender segregation among different occupations and economic sectors is well-known, and significant differences in the distribution of occupational exposure patterns by gender have been detected [4]. A recent cross-sectional study suggests a number of factors influencing prevalence and level of exposure to carcinogenic, mutagenic and reprotoxic agents, gender included [5].

Different working conditions, as well as the absorption rate, metabolism and bioavailability of chemicals are likely to occur in women than in men [6]. A different cellular response to oxidative stress between men and women in cancer susceptibility was also recently hypothesized in a study conducted by Ali and colleagues, that concluded raising the question whether the carcinogens classifications should be gender specific [7]. Another matter of concern is represented by concurrent exposure to carcinogenic chemicals in several activity sectors. The existence of multiple exposure patterns should be taken into great account, both at individual and group level, when assessing occupational risk factors [8].

In Italy, since 1996 employers are required to record in a register the workers’ exposure to chemicals classified as carcinogen by the EU, with the aim of monitoring and controlling the exposure risk [9]. This register is transmitted both to inspection authorities and, for epidemiologic and research purposes, to the Italian workers’ compensation authority (INAIL).

The aim of this study is to describe levels and patterns of exposure to the most common cancer-causing agents among Italian workers, focusing on gender differences.

## Methods

### Data gathering

The most common occupational carcinogens were identified by selecting data from the National System on Occupational Exposure to Carcinogens (SIREP) instituted at the INAIL. The minimum number required to select the carcinogenic agents was set to 100 measurements. All data on exposure measurements over the period 1996–2015 were taken into consideration. SIREP is a relational database whose design and contents have been fully described elsewhere [9]. In brief, Italian law requires that employers collect data on workers’ exposures to carcinogens and report this information to INAIL, updating data every three years. The reporting is mandatory for carcinogens classified as 1A and 1B by the new Globally Harmonized System of classification (GHS) currently adopted by the EU (1A, known to be carcinogenic; 1B, presumed to be carcinogenic). Employers are required to fill in a standardized form which includes personal and occupational data of exposed employees, the carcinogenic agents to which they are exposed and the exposure level (in term of intensity, frequency and duration), besides the economic activity sector and the size of firm’s workforce. One or more exposure measurements are recorded for each worker and work period. Employers are responsible for the exposure measurement procedures and air sampling methods, to be carried out in accordance with European standards which provide technical guidance to implement an air monitoring strategy [10].

### Data selection and classification

Overall, 40 most common carcinogens were identified in the SIREP database. Some of these agents were further arranged in groups, leading to 21 the groups/agents selected for the study. Exposure measurements refer to an 8-h working period, and the exposure intensity was classified in three categories by comparison with the time-weighted average threshold limit value (TWA-TLV) proposed by the American Conference of Governmental Industrial Hygienists (ACGIH) for each of the selected agents, following a methodological approach already applied for a similar study [11, 12]. One carcinogen agent (residual heavy fuel oil) was excluded from the analysis due to the absence, at the time of the study, of a specific TWA-TLV. In detail, high intensity (H) was considered for average concentration values higher than half the TWA-TLV, medium intensity (M) for values between half and a quarter of the TWA-TLV, and low intensity (L) for values equal or lower than a quarter of the TWA-TLV. The exposure-related variables selected for descriptive and risk analysis were: carcinogenic agent/group, occupational group, economic activity sector and workforce size. Occupational group was coded using the international standard classification of occupations (ISCO-88) at the major group level (one-digit code); activity sector was categorized using the international statistical classification of economic activities (NACE rev. 1) at the division level (two-digit code); and workforce size was categorized in 5 classes: 1–9, 10–19, 20–49, 50–99, ≥ 100 workers. Pearson’s chi-square (χ^2^) was used to test for differences in the distribution of categorical variables by gender. In order to more directly describe the proportion of women in each group, the male to female (M/F) ratio was calculated.

### Logistic regression analysis

In order to study the association between gender and carcinogen exposure level (classified in L, M, and H intensity, as described above), logistic regression models were applied in the whole dataset by introducing, one at time separately, each of the exposure-related variables (namely, carcinogenic agent/group, occupational group, economic activity sector and workforce size). Each model was corrected for age at exposure taken as a continuous variable (workers aged between 20 and 75 years old). Adjusted odds ratios (ORs) with 95% confidence intervals (CI) were calculated for each exposure-related variable.

### Co-exposures analysis

To explore the patterns of concurrent exposures to multiple carcinogen agents, a cluster analysis based on the SPSS two-step clustering method was performed, using the Bayesian information criterion (BIC) as clustering method and the log-likelihood as distance measure. In brief, this method consists of two steps: in the first step a sequential clustering approach is used to create sub-clusters based on the distance criterion; subsequently (second step) sub-clusters resulting from the first step are grouped into clusters through the agglomerative hierarchical clustering method. To determine the number of clusters, the BIC for each number of clusters within a specified range is calculated, considering the smallest BIC value as the best cluster solution [13]. The results are presented in gender-combined and gender-separated group comparisons. The data were collected routinely as an institutional activity and were analysed anonymously using IBM SPSS Statistics v. 22 (IBM Corp., Armonk, NY, USA).

## Results

### Descriptive statistics

A total of 166,617 exposure measurements of the 21 carcinogenic agents/groups selected were available for the analysis (Table 1). Regarding the intensity of exposure, 75% of measurements were classified in the low level group (L), 16% at the medium level (M) and the remaining 9% at high level (H). Most exposures were in men (90%), but a relevant number of exposure measurements were recorded in female workers too (15093). Age distribution did not differ by gender, and most workers were aged 20–34 years (37%) and 35–44 years (33%). The highest proportion of workers was exposed to hardwood dust, 74% for women and 51% for men. Other relevant exposures in female workers were to chromium VI and nickel compounds (7%, 4% respectively), benzene (5%) and formaldehyde (1%). The lowest male to female ratio (M/F) was detected among workers exposed to vinyl chloride monomer (2.5) whereas the highest was for asbestos (303.8) (Table 2). The occupational group best represented in the selected data was craft and related trades workers (51% of exposures), while the lowest M/F ratios were among clerks (3.9) and managers and professionals (5.1). The manufacture of furniture was the economic sector that counted more exposures in men workers (23%), but it was the second most common sector of exposure among women (27%) after the manufacture of wood and products of wood and cork (43%). The highest M/F ratios were found in the construction and in the manufacture of coke and refined petroleum products, the lowest in the wood and rubber and plastics industries. Large firms (≥100 workers) reported the highest proportion of exposures (36%) and the lowest M/F ratio (7.6).

**Table 1:** Most common occupational carcinogenic agents/groups in SIREP database and number of measurements by exposure level

Agent/group	CAS n°	Agent in group	L	M	H
Acrylamide	79–06-1		1920	376	151
Acrylonitrile	107–13-1		950	30	–
Benzene	71–43-2		18,674	805	682
1,3-butadiene	106–99-0		2775	66	36
Vinyl chloride monomer	75–01-4		775	–	217
1,2-dichloroethane	107–06-2		654	36	55
Formaldehyde	50–00-0		1077	207	374
Epichlorohydrine	106–89-8		702	40	39
Hydrazine	302–01-2		512	115	169
Ethylene oxide	75–21-8		587	67	31
Propylene oxide	75–56-9		666	42	29
Trichlorethylene	79–01-6		931	70	85
Dinitrotoluene (isomers mixture)	25321-14-6		687	1	17
Hardwood dust	–		56,836	21,588	10,030
Refractory ceramic fibers	142844-00-6		248	72	116
Asbestos	12172-73-5	Amosite	698	385	31
	12001-29-5	Chrysotile	3540	706	196
	12001-28-4	Crocidolite	525	509	24
	132207-32-0	Asbestos	205	134	58
Silica crystalline	14808-60-7	Quartz	500	218	104
	14464-46-1	Cristobalite	258	46	451
PAHs	56–55-3	Benzo[*a*]anthracene	1591	13	48
	50–32-8	Benzo[*a*]pyrene	1566	105	120
	205–99-2	Benzo[*b*]fluoranthene	1313	49	18
	205–82-3	Benzo[*j*]fluoranthene	863	49	42
	207–08-9	Benzo[*k*]fluoranthene	1269	49	18
	53–70-3	Dibenz[*a,h*]anthracene	914	14	17
	192–97-2	Benzo[*e*]pyrene	1138	–	5
	218–01-9	Chrysene	1360	13	5
	–	Particulate PAH	2501	92	206
Chromium VI and compounds	1333–82-0	Chromium trioxide	7005	232	607
	7789–00-6	Potassium chromate	231	–	2
	7789–06-2	Strontium chromate	281	59	137
	7440–47-3	Chromium	3494	17	78
Nickel and compounds	–	Insoluble compounds	867	14	22
	1313–99-1	Nickel(II) oxide	1538	118	73
	7786–81-4	Nickel(II) sulfate	1073	233	111
	7440–02-0	Nickel	3315	11	25
	7791–20-0	Nickel(II) chloride	752	48	80
Cadmium	7440–43-9		620	12	56
*Overall*			*125,411*	*26,641*	*14,565*

*PAH* Polycyclic Aromatic Hydrocarbon, *L* low level, *M* moderate level, *H* high level

**Table 2:** Gender differences in the risk of having higher occupational exposure levels by exposure-related variables

Variable	Description	Total	Males (ref.)	Females	M/F Ratio	OR (95% CI) for medium level^a^	OR (95% CI) for high level^a^
*Agent/Group* (χ^2^ *p* value < 0.01*)*							
79–06-1	Acrylamide	2447	2337	110	21.2	1.59 (1.00 to 2.51)†	0.29 (0.07 to 1.19)
107–13-1	Acrylonitrile	980	859	121	7.1	0.23 (0.03 to 1.68)	–
71–43-2	Benzene	20,161	19,459	702	27.7	0.56 (0.34 to 0.93)†	0.82 (0.53 to 1.28)
106–99-0	1,3-butadiene	2877	2756	121	22.8	–	–
75–01-4	Vinyl chloride monomer	992	708	284	2.5	–	1.62 (1.17 to 2.24)†
107–06-2	1,2-dichloroethane	745	666	79	8.4	0.79 (0.29 to 2.56)	–
50–00-0	Formaldehyde	1658	1446	212	6.8	0.94 (0.56 to 1.58)	2.52 (1.83 to 3.46)†
106–89-8	Epichlorohydrine	781	749	32	23.4	–	–
302–01-2	Hydrazine	796	786	10	78.6	–	0.41 (0.05 to 3.29)
75–21-8	Ethylene oxide	685	598	87	6.9	1.03 (0.49 to 2.17)	0.41 (0.10 to 1.76)
75–56-9	Propylene oxide	737	668	69	9.7	1.14 (0.39 to 3.33)	3.40 (1.29 to 8.94)†
79–01-6	Trichlorethylene	1086	1025	61	16.8	1.71 (0.65 to 4.49)	6.28 (3.33 to 11.9)†
25321-14-6	Dinitrotoluene (isomers mixture)	705	696	9	77.3	–	–
–	Hardwood dust	88,454	77,234	11,220	6.9	0.70 (0.66 to 0.74)†	0.84 (0.79 to 0.90)†
142844-00-6	Refractory ceramic fibers	436	397	39	10.2	0.09 (0.01 to 0.67)†	0.12 (0.03 to 0.49)†
Various	Asbestos	7011	6988	23	303.8	1.96 (0.84 to 4.58)	–
Various	Silica crystalline	1577	1547	30	51.6	0.76 (0.30 to 1.90)	0.06 (0.01 to 0.47)†
Various	PAHs	13,378	13,076	302	43.3	–	0.07 (0.01 to 0.50)†
Various	Chromium (VI) and compounds	12,143	11,115	1028	10.8	0.69 (0.43 to 1.11)	1.47 (1.17 to 1.83)†
Various	Nickel and compounds	8280	7748	532	14.6	0.49 (0.29 to 0.85)†	1.56 (1.06 to 2.29)†
7440–43-9	Cadmium	688	666	22	30.3	–	6.78 (2.67 to 17.2)†
*Occupational group* (χ^2^ p value < 0.01*)*							
1&2	Managers and professionals	1338	1119	219	5.1	0.09 (0.01 to 0.65)†	0.40 (0.14 to 1.15)
3	Technicians and associate professionals	5328	4789	539	8.9	0.38 (0.25 to 0.59)†	0.92 (0.60 to 1.42)
4	Clerks	1418	1127	291	3.9	0.86 (0.56 to 1.32)	0.11 (0.04 to 0.30)†
5	Service and sales workers	5175	4766	409	11.7	0.68 (0.43 to 1.05)	0.49 (0.28 to 0.85)†
6	Craft and related trades workers	85,039	76,892	8147	9.4	0.89 (0.84 to 0.94)†	1.10 (1.02 to 1.18)†
7	Plant, machine operators and assemblers	62,006	56,902	5104	11.1	1.12 (1.03 to 1.22)†	1.61 (1.45 to 1.79)†
8	Elementary occupations	6313	5929	384	15.4	1.72 (1.31 to 2.25)†	1.40 (1.03 to 1.91)†
*Workforce size* (χ^2^ p value < 0.01*)*							
a)	1 to 9 workers	23,039	21,320	1719	12.4	1.05 (0.93 to 1.18)	0.84 (0.72 to 0.99)†
b)	10 to 19 workers	23,681	21,953	1728	12.7	1.62 (1.44 to 1.81)†	1.45 (1.25 to 1.68)†
c)	20 to 49 workers	38,231	35,544	2687	13.2	0.99 (0.88 to 1.10)	1.02 (0.90 to 1.16)
d)	50 to 99 workers	21,757	19,725	2032	9.7	1.13 (1.00 to 1.28)†	1.98 (1.70 to 2.31)†
e)	> = 100 workers	59,909	52,982	6927	7.6	0.80 (0.73 to 0.87)†	1.62 (1.48 to 1.78)†
*Economic sector*^*b*^ (χ^2^ p value < 0.01)							
20	Manufacture of wood and wood products	36,599	30,097	6502	4.6	0.80 (0.75 to 0.86)†	0.92 (0.85 to 1.01)
23	Manufacture of refined petroleum products	12,213	12,061	152	79.3	2.97 (0.71 to 12.4)	–
24	Manufacture of chemicals	12,585	12,036	549	21.9	0.82 (0.60 to 1.12)	1.20 (0.82 to 1.76)
25	Manufacture of rubber and plastic products	1486	986	500	2.0	0.27 (0.14 to 0.50)†	0.74 (0.56 to 0.98)†
26	Manufacture of other non-metallic products	1043	963	80	12.0	0.21 (0.08 to 0.52)†	1.05 (0.58 to 1.91)
28	Manufacture of fabricated metal products	10,320	9352	968	9.7	0.93 (0.70 to 1.24)	2.36 (1.92 to 2.89)†
29	Manufacture of machinery and equipment nec	5093	4567	526	8.7	0.15 (0.08 to 0.28)†	0.12 (0.05 to 0.30)†
34	Manufacture of motor vehicles	1425	1303	122	10.7	0.95 (0.49 to 1.82)	0.27 (0.10 to 0.74)†
35	Manufacture of other transport equipment	8536	8198	338	24.3	0.32 (0.23 to 0.44)†	0.17 (0.08 to 0.34)†
36	Manufacture of furniture; manufacturing nec	38,097	34,069	4028	8.5	0.61 (0.56 to 0.67)†	0.83 (0.75 to 0.92)†
45	Construction	13,720	13,685	35	391.0	2.68 (1.18 to 6.13)†	2.95 (1.02 to 8.57)†
50	Repair of motor vehicles; retail sale of fuel	2828	2608	220	11.9	1.01 (0.48 to 2.10)	0.45 (0.24 to 0.85)†
51	Wholesale trade and commission trade	1673	1591	82	19.4	0.10 (0.01 to 0.72)†	0.86 (0.42 to 1.76)
52	Retail trade, repair of household goods	2435	2325	110	21.1	0.86 (0.49 to 1.54))	5.03 (2.70 to 9.35)†
74	Other business activities	1620	1534	86	17.8	0.27 (0.11 to 0.68)†	0.24 (0.06 to 0.98)†
90	Sewage and refuse disposal, sanitation	4707	4609	98	47.0	0.08 (0.01 to 0.57)†	–
	*Overall*	*166,617*	*151,524*	*15,093*	*10.0*	*0.96 (0.92 to 1.01)*	*1.20 (1.13 to 1.27)†*

^a^Low level is the reference group; †Significant at *p* = 0.05 level; OR: Odds ratios; 95%CI: 95% confidence interval; ^b^only main sectors are showed; nec: not elsewhere classified; Elementary occupations consist of simple and routine tasks which mainly require the use of hand-held tools

### Logistic regression analysis

In the total dataset, women were more likely to be exposed to high levels than men (OR = 1.20; 95%CI: 1.13–1.27) regardless of the carcinogenic agent of exposure. Gender differences in the risk of having higher occupational exposure levels by exposure-related variables are shown in Table 2. After adjusting for age at exposure, female workers were more likely to have high exposure levels to formaldehyde, monomer vinyl chloride, propylene oxide, trichloroethylene, cadmium, nickel and chromium IV compounds than males. Conversely men were more likely to be exposed to medium and/or high levels of hardwood dust, crystalline silica, refractory ceramic fibres, benzene and polycyclic aromatic hydrocarbons (PAHs). For some agents, such as 1,3-butadiene, asbestos, etc., it was not possible to evaluate the OR for both exposure levels (medium and high intensity) due to data paucity. The risk of having high or medium exposure levels with respect to the low level was significantly higher in women employed as blue collars (occupational group codes from 6 to 8), hired either in small (10–19 workers) or medium-large firms (> 50 workers). Higher exposure levels among women were more often recorded in firms belonging to the construction, the manufacture of fabricated metal products and the retail trade (mainly furniture and hardware), while lower levels were detected in a large number of sectors, including the manufacturing of rubber and plastic products, machinery and equipment, motor vehicles, other transport equipment and furniture.

### Co-exposures analysis

**Fig. 1:**
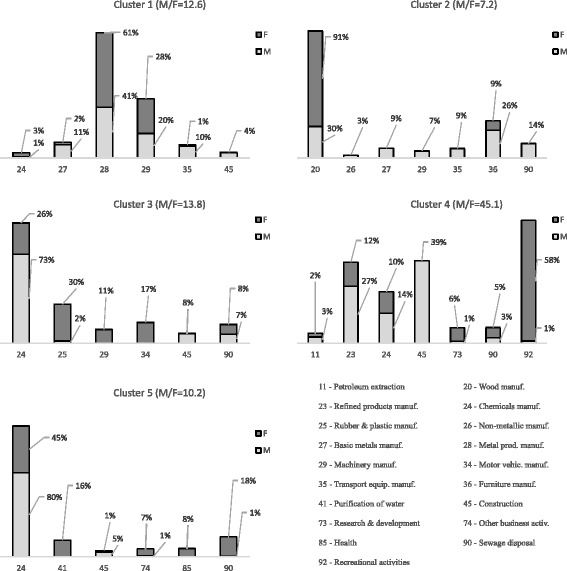
Distribution (%) of co-exposed workers in each identified gender-separated cluster by the most common activity sectors

A maximum of 12 agents with simultaneous exposures was detected in the dataset, and more than 13% of workers were found exposed to two or more agents. In a very little proportion of cases (3.3%) exposures to multiple chemicals occurred in different work periods during worker’s activity. By using the two-step cluster analysis, five main clusters were identified in both genders together and separately. The distribution of exposure prevalence to each carcinogen among clusters by gender is reported in Table 3. The corresponding index of cohesion and separation of clusters found was good (silhouette index = 0.5 for both genders together and for men, 0.7 for women). The distribution of co-agents within the identified clusters was practically overlapping in both genders, with exception of clusters n° 2 and 3. Most relevant concurrent exposures occurred between nickel and chromium VI compounds (cluster n° 1; 12,520 exposure measurements of which 919 in women), and among benzene, 1,3-butadiene and PAHs (cluster n° 4; 18,012 exposure measurements of which 391 in women). Other main co-exposure patterns were identified in workers exposed simultaneously to hardwood dust and formaldehyde (cluster n° 2), silica crystalline and refractory ceramic fibers (cluster n° 2, in men only); acrylamide, acrylonitrile, and vinyl chloride monomer (cluster n° 3); ethylene oxide, propylene oxide, epichlorohydrine, trichloroethylene, and dinitrotoluene (cluster n° 5). The M/F ratio varied from 7.2 in cluster n° 2 to 45.1 in cluster n° 4 (Fig. 1).

**Table 3:** Distribution (in %) of the prevalence of each carcinogenic agent/group among identified clusters by gender

CAS n°	Agent/Group	Cluster n° 1			Cluster n° 2			Cluster n° 3			Cluster n° 4			Cluster n° 5		
		All	M	F	All	M	F	All	M	F	All	M	F	All	M	F
79–06-1	Acrylamide	0	0	0	4	0	0	96	100	80	0	0	0	0	0	20
107–13-1	Acrylonitrile	0	0	0	0	0	0	76	74	76	0	0	6	24	26	18
71–43-2	Benzene	0	0	0	1	6	0	28	23	12	61	61	59	11	11	29
106–99-0	1,3-butadiene	0	0	0	2	0	0	29	42	53	57	47	36	11	12	11
75–01-4	Vinyl chloride monomer	0	0	0	1	2	0	61	60	28	0	0	0	38	38	72
107–06-2	1,2-dichloroethane	0	0	0	1	7	0	68	61	0	0	0	3	31	33	97
50–00-0	Formaldehyde	0	0	6	91	83	71	9	17	18	1	0	1	0	0	4
106–89-8	Epichlorohydrine	0	0	0	5	5	0	36	34	62	0	0	0	60	62	39
302–01-2	Hydrazine	0	0	0	2	2	0	98	98	100	0	0	0	0	0	0
75–21-8	Ethylene oxide	0	0	0	0	0	0	42	41	0	0	0	29	58	59	71
75–56-9	Propylene oxide	0	0	0	0	0	0	34	34	0	0	0	0	66	67	100
79–01-6	Trichlorethylene	0	0	41	15	22	0	22	12	0	0	0	0	63	66	59
25321-14-6	Dinitrotoluene (isomers mixture)	0	0	0	0	0	0	6	7	0	1	0	0	93	93	100
–	Hardwood dust	0	0	1	77	86	91	23	14	0	0	0	7	0	0	1
142844-00-6	Refractory ceramic fibers	0	0	0	100	100	0	0	0	0	0	0	0	0	0	0
Various	Asbestos	0	0	0	98	99	0	2	2	0	1	0	100	0	0	0
Various	Silica crystalline	0	0	0	100	100	0	0	0	0	0	0	0	0	0	0
Various	PAHs	0	0	0	9	10	0	14	26	0	62	47	91	16	16	9
Various	Chromium (VI) and compounds	75	75	83	15	17	0	4	1	0	7	7	8	0	0	9
Various	Nickel and compounds	71	71	89	15	15	0	1	1	4	13	13	3	0	0	3
7440–43-9	Cadmium	0	0	0	94	100	0	6	0	100	0	0	0	0	0	0

All: both genders together, *M* Males, *F* Females

The sector of economic activity best represented in co-exposure clusters was chemical industry (mainly in clusters n° 3 and 5). Co-exposures were also found in the manufacture of fabricated metal products and of machinery and equipment (cluster n° 1), in the wood industry and manufacture of furniture (cluster n° 2), and in the sector of refined petroleum products (cluster n° 4). In Fig. 1 is shown the distribution, in percentage, of co-exposures in the most common activity sectors within each identified cluster, separately by gender. As regard the distribution of occupational groups among clusters, some differences were observed: in cluster n° 1 the majority of men were craft and related trades workers, while the most common occupation for women was plant, machine operator and assembler; in clusters n° 4 and 5 the main occupation was plant, machine operator and assembler in men, while in women it was clerk and technician. In terms of workforce, the most relevant concurrent exposures (in both genders) were found in large firms (> 100 workers), with the exception of clusters n° 2 and 4 in which a relevant number of women co-exposed to more than one agent was found in the smaller firms (50–100 workers).

## Discussion

This study highlights the peculiarities about current exposure to carcinogenic agents among female workers in Italy. Its main strength is to rely on data from a national registry of occupational exposures to carcinogens covering the period 1996–2015, which made possible to collect and analyse a large number of exposure measurements in the female workforce coming from a great variety of economic sectors, over the whole Italian territory. Moreover, this study applied cluster analysis in order to identify the main concurrent exposures among the most common occupational carcinogens and their distribution by gender. As a whole, our findings provide useful information that can be also applicable to other European and to high-income countries sharing with Italy a similar economic structure. This information may contribute to the international discussion dealing with the implementation of prevention strategies for occupational cancer, and with the need of taking into account the gender in the occupational safety and health (OSH) management as well.

Women represent a small but still remarkable proportion of workers exposed to occupational carcinogens, in line with estimates performed in other developed countries [3]. Our study confirms the presence of a clear gender segregation by occupation, as found by an analysis conducted in 2007 on working conditions in the EU [14]. Moreover, unexpectedly, being female is associated in our study with an increased risk of having a high level of exposure, considering the selected carcinogens as a whole (OR = 1.20; 95%CI: 1.13–1.27). More specifically, a number of gender differences were appreciated both in carcinogenic agents involved and in exposure levels. However, because of limited data for some agents, the reported results should be considered with caution.

The most common exposure recorded in both genders was indeed to hardwood dust, occurring mainly in the wood industry and in the manufacture of furniture where was often associated with exposure to formaldehyde. Previous Italian estimates of workers exposed to hardwood dust accounted for 38,820 men and 6072 women exposed, taking into account only the sector of manufacture of furniture [15]. According to WOODEX study, about 3.6 million workers were exposed to inhalable wood dust in EU-25 (351,000 in Italy) in 2000–2003 years, but no gender-specific estimates have been provided [16]. In our study, exposure levels to hardwood dust were higher in male workers, while women had higher levels of exposure to formaldehyde, a chemical recently added in the carcinogens list of EU. A study based on SIREP data has shown higher mean exposure levels to formaldehyde in sectors with a higher proportion of women exposed such as wood industry and healthcare [17]. The concurrent occupational exposure to hardwood dust and formaldehyde emerges as a critical issue, involving, according to our data, a relevant number of women too, mainly in small-size enterprises. Considering that both these chemicals have been associated, with a synergic action, to an increased risk of sino-nasal cancers [18], specifically targeted prevention policies and epidemiological surveillance programs need to be implemented. The occurrence of sino-nasal cancer in women is a great concern in Italy according to the last estimates of the Italian sino-nasal cancer register, that evaluated an incidence rate of 0.24 (per 100,000 person-years) for women in the period 2010–2014 with a M/F ratio equal to 2.7 [19].

The co-exposure to nickel and chromium VI compounds was commonly found, in both genders, in the manufacturing of metal products and in metalworking industry, while in men it was observed mainly in the sectors of metallurgy and manufacturing of transport equipment. The higher levels of exposure recorded in women for these metals could be almost in part due from the different occupations of exposed workers by gender, being women more often plant or machine operators (82% of exposed women to these metals). As regard the occupation in the overall exposed population of our study, women were prevalent among clerks (M/F ratio = 3.9), while men constituted the majority of production workers and machine operators (M/F = 11.1). However, women working as machine operators were more likely to be exposed to medium or high levels than men (OR = 1.12 and 1.61 respectively). The possibility that further factors other than occupation, such as workstation, task or skills, can cause overexposure of female workforce in these setting deserves to be investigated by sector-specific surveys. Information about workers’ education or use of personal protection equipment is not included in the source of our data. The findings of our study, however, suggest the need to implement protective measures for female workers engaged in the manufacture of fabricated metal products.

Multiple exposures to carcinogens were common, in both genders, in the manufacturing of chemicals with two major exposure clusters being identified by our analysis (cluster n° 3 and 5). However, some co-exposures were also recorded in other activity sectors, showing some substantial differences in the distribution by gender. In particular, women were in proportion more often co-exposed to acrylamide, acrylonitrile and vinyl chloride monomer in the manufacturing of rubber and plastics, while men resulted mostly exposed in the sewage disposal sector for their co-exposures to ethylene oxide, propylene oxide, epichlorohydrine, trichloroethylene and dinitrotoluene. Despite lower exposure levels respect to men, the relevant number of exposed women in some of these sectors, such as rubber and plastics industry, must be taken into account when programming health surveillance plans aimed to prevent occupational cancer.

The construction industry and the manufacture of refined petroleum products were confirmed to be male-dominated sectors for the exposure to carcinogenic agents, as already observed in a similar study in New Zealand [4]. Male workers in the construction sector had more co-exposure patterns (clusters n° 1, 3, 4 and 5), but the few women employed in this sector were more likely to be exposed to medium and high levels than men (OR = 2.68 and 2.95 respectively). Co-exposures to benzene, 1,3-butadiene and PAHs (cluster n° 4) are well known among oil refinery workers, even if exposure levels are currently low [20, 21]. However, a similar pattern of exposure can also occur in the service sector, with a share in women too as reported in a previous study [17]. In the present study, this co-exposure pattern among women (cluster n° 4) was found mainly in the sector of gambling and betting activities, for exposures coming from secondhand smoke. In a recent study, after mining and construction, the industry sector with the highest observed prevalence rate of non-smoking workers exposed to workplace secondhand smoke were the arts, entertainment, and recreation activities [22]. Environmental tobacco smoke represents one of the most common exposures experienced by women in the CAREX studies, but currently exposure information systems are unlikely to record in a representative way this kind of exposure at workplace [3], and thus any further interpretation is precluded.

The main limitations of SIREP database are the inhomogeneous territorial coverage and the under-representation of some economical activities, as already underlined in previous studies [15, 17, 23]. Data collection and reporting for the SIREP database are under the responsibility of the employer, and we observed that the number of exposure measurements differed by industrial sector and workforce size. Data referring to the exposure level classes, as identified in the methods section (L, M, H), were in large quantities for some activities/occupations but limited for others. For this reason the reported results should be considered with caution. Moreover, an underreporting of data for smaller firms is likely because of a lower commitment to workers’ health and safety by this set of firms [24]. The possibility that firms which do not record or transmit data on exposures have higher exposure levels, might have affected our estimates. Uncertainty may have been also introduced as a result of differences in air sampling, analytical procedures, sample collection methods (personal or stationary) and data classification. In order to increase the precision of analysis, a number of 100 measurements was set as the minimum number required to select the carcinogenic agents. The choice of use the ACGIH TLVs to classify the exposure intensity stems from the incompleteness of occupational exposure limits established by the EU legislation for carcinogens, leading to wide differences across Member States. In addition, the ACGIH TLVs are largely used in practice as a reference in the chemical risk assessment in Italian workplaces. Moreover in such a way, a better comparison with other international studies on exposure assessment is warranted. Other problems inherent in the use of administrative sources as SIREP are the original purpose of data collection (e.g. complaint, compliance, research, etc.), changes in measurement techniques and variability in environmental conditions, that may distort exposure measurements if missing [25]. Finally, the cluster analysis performed (SPSS two-step method) to investigate co-exposures was adopted since designed to be particularly suited for handling large datasets, and it has the advantage of not requiring to set a priori the number of clusters. On the other hand, it shares the limits of hierarchical methods, such as not taking into account the differences between relevant and irrelevant variables and being very sensitive to outliers.

## Conclusions

This study shows significant disparities in the prevalence and level of occupational exposures to carcinogens among female and male workers in the Italian workforce. Moreover, in certain occupational settings women, compared to men, were more likely to be exposed to high levels of carcinogens. The overall findings provide useful information both for decision making in prevention policies and for programming epidemiological studies on occupational cancer in the female workforce. Likewise, an accurate carcinogenic risk assessment based on concentration levels and co-exposure patterns can help to address prevention and health promotion plans in the workplaces.

## Abbreviations

ACGIH: American Conference of Governmental Industrial Hygienists
BIC: Bayesian information criterion
CI: Confidence Intervals
EU: European Union
GHS: Globally Harmonized System
H: High intensity
INAIL: Italian Workers’ Compensation Authority
ISCO: International Standard Classification of Occupations
L: Low intensity
M: Medium intensity
M/F: Male/Female
NACE: Nomenclature of economic activities in the European community
OR: Odds Ratios
PAH: Polycyclic Aromatic Hydrocarbons
SIREP: Italian Occupational Exposures Database
TWA-TLV: Time-Weighted Average Threshold Limit Value
